# Electrocautery, bronchoscopic biopsy and hemorrhage

**DOI:** 10.4103/1817-1737.74282

**Published:** 2011

**Authors:** Andrew R. L. Medford

**Affiliations:** *North Bristol Lung Centre, Southmead Hospital, Westbury-on-Trym, Bristol, UK. E-mail: andrewmedford@hotmail.com*

Sir,

The potential utility of bronchoscopic cryobiopsy in your recently published study by Aktas *et al*. is noted.[1] A previous theoretical cost analysis also supports the use of cryobiopsy in achieving cost savings by reducing the rate of nondiagnostic biopsies.[2] One ongoing concern is of hemorrhage following bronchoscopic biopsy, whether forceps or cryobiopsy. In the study by Aktas *et al*., adrenaline, cold saline, or argon plasma coagulation was required in between 21% and 25% of cases.[1] A method of reducing postbiopsy hemorrhage would therefore be welcome without affecting the histology rate. Electrocautery coagulates, vaporises or cuts tissue (depending on the power setting) by using alternating current at high frequencies to generate heat.[3] It is used in hot biopsy at colonoscopy, and in controlling hemoptysis at bronchoscopy.[4]

We utilized electrocautery *via* a flexible bronchoscope in 34 consecutive patients with vascular endobronchial tumors which might have been predicted to bleed more profusely following the biopsy. Electrocautery was applied *via* an Olympus BFP240 insulated video bronchoscope using an Olympus PSD-20 Electrosurgical unit monopolar circuit with an MH-551 foot switch, 350 kHz output, an Olympus CD-6C-1 coagulation ball type probe with power settings at 5 W (cutting) and 15 W (coagulation). Pulses of electrocautery were only applied for 0.5 s only cauterizing 25–50% of the tumor to minimize histological damage, but sufficient to be more confident of hemostasis. Following this, biopsies were taken with standard biopsy forceps. No significant bleeding occurred, and there were no complications relating to the use of electrocautery. All procedures were carried out under local anesthesia as per conventional flexible bronchoscopy. The diagnostic yield was 88.2% (30 out of 34 patients) which compared favorably with the histological yield for nonelectrocauterized biopsies (89.1%, 33 out of 37 previous consecutive patients) and well above the minimum standard of 80% national guidelines from the British Thoracic Society.[5]

In summary, controlled bronchoscopic electrocautery shows promise as a mode of reducing hemorrhage following bronchoscopic forceps biopsy without affecting the diagnostic yield although these findings require validation in suitably powered prospective studies and also assessing its possible role following cryobiopsy.
